# Leucocytozoonosis in Domestic Birds in Southwestern Iran: An Ultrastructural Study

**Published:** 2013

**Authors:** O Dezfoulian, M Zibaei, H Nayebzadeh, M Haghgoo, AN Emami-Razavi, K Kiani

**Affiliations:** 1 Department of Pathobiology, Faculty of Veterinary Medicine, Lorestan University, Khorram Abad, Iran; 2 Department of Parasitology and Mycology, School of Medicine, Lorestan University of Medical Sciences, Khorram Abad, Iran; 3 Office of Agriculture Insurance Found, Lorestan Province, Khorram Abad, Iran; 4 Department of Pathology, School of Medicine, Tehran University of Medical Sciences, Tehran, Iran; 5 Department of Pharmacology, Faculty of Veterinary Medicine, University of Tehran, Tehran, Iran

**Keywords:** *Leucocytozoon*, leukocytosis, Protozoa, Native poultry, Iran

## Abstract

**Background:**

Leucocytozoonosis is a disease of birds caused by obligate intracellular protozoa of the genus *Leucocytozoon*. We determined the prevalence of *Leucocytozoon* spp. using light and transmission electron microscopy in domestic birds in southwest of Iran.

**Methods:**

A total of 825 blood smears from 275 birds were examined for presence of infection. The structure morphology of *Leucocytozoon* spp. was studied using light and electron microscopy.

**Results:**

Forty-four (16.0%) of the birds were positives for *Leucocytozoon*. The detected parasite were found in 14 chickens (5.1%), 12 geese (4.3%), 10 ducks (3.6%), and 8 turkeys (2.9%). The majority of the records were from the northeastern regions.

**Conclusion:**

Leucocytozoonosis are distributed in the Lorestan province bird population and electron microscopy can resolve the problem to distinguish between similar species of *Leucocytozoon*.

## Introduction

*Leucocytozoon* is a haemosparasite of the apicomplexa phylum considered to be host-specific at the family level and is found in birds worldwide (1).

Leucocytozoonosis is a disease of birds caused by obligate intracellular protozoa of the genus *Leucocytozoon*. The disease is transmitted by bite of Simuliidae (black flies) and causes severe anemia, weight loss and death in susceptible birds (2). Young birds are more susceptible than adults, and the most serious mortality generally occurs within the first few weeks of hatching (3). There are many *Leucocytozoon* species may be putatively involved: *L. simondia* (ducks and geese), *L. smithi* (turkey), *L. bonasae* (grouse and ptarmigan) and L. *marchouxi* (pigeons and doves) (2, 4).

*Leucocytozoon* affects circulating leucocytes and erythrocytes as well as tissue macrophages and endothelial cells, where in the latter it creates large tissue schizonts up to 700 µm in diameter (5). Although the ornithological fauna of Lorestan province, Iran may be highly diverse, the blood parasites of this avifauna have had only limited study. *Aegyptianella* spp. is one of the common obligate intracellular parasites affecting poultry in the area (6).

This study had the aims of evaluating the prevalence of leucocytozoonosis in the bird populations in several areas of the southwest of Iran using light and electron microscopy.

## Materials and Methods

### Study area

This study was conducted in the Lorestan Province of the southwest Iran during the April and September 2011. Climatically, the province can be divided into three parts: the mountainous regions (north areas), experience cold winters and moderate summer. In the central region, the spring season beings from mid-February and lasts till about mid-May. The south areas have hot summers and relatively moderate winters.

### Sample size

Due to the lack of any information about the prevalence of *Leucocytozoon* in birds in the province, we assumed for large sampling procedures an infection prevalence of around 30 percent sample size calculation was based upon this prevalence, accepting desired absolute precision of 5% and a confidence interval of 95%. Consequently, about 275 birds (chickens, n = 99; geese, n = 87; turkeys, n = 50; and ducks, n = 39) were examined to judge this prevalence estimation.

### Ethical consideration

Approval of the study protocol was received from the Ethical Review Board of Lorestan University.

### Blood collection and smears

All birds selected for investigation were clinically examined and data were recorded, and then released after taking a small amount of blood (one ml) from brachial wing vein of each bird subjected to study. The blood smears were air-dried, fixed in methanol and stained in 3% Giemsa solution diluted in phosphate buffer saline (pH 7.2) for 20 min. The stained slides were examined using an Olympus CX31 microscope and oil immersion lens at 100×, 200×, 400×, and 1000× power field for 20-25 min. Presence and intensity of parasites was recorded.

### Electron microscopy study

Infected blood was provided by fresh peripheral blood smears and their infections were reconfirmed. Some collected blood drops were clotted at room temperature and prefixed with 2.5% glutaraldehyde solution (TAAB Laboratories-3 Minerwa, Calleva park, Aldermaston, Berks, RG78NA, England-EM grade, Batch No.58030) in 0.1 M phosphate buffer saline (pH 7.2) for 120 min at 4 °C for preparation of transmission electron microscopy (TEM). Then, the samples were washed three times in the phosphate buffer saline (10 min, each time), post-fixed in 1% osmium tetroxide solution (Batch No. 48290) in the same buffer at room temperature for 120 min. Then washed in the phosphate buffer saline (10 min), dehydrated in ascending alcohol series and finally embedded in TAAB resin (Berks, RG74QW, England, Data Sheet No. 3), polymerized in 60°C for 48 h. 50 nm ultra-thin sections were then prepared by LKB ultratome 4801A (LKB-producer AB-Stockholm 12-Sweden) and double stained with 20% uranyl acetate solution (B.D.H. Laboratory Chemical Division, England, No. 0148860) in pure methanol (E. Merck, D-6100 Darmstadt, F-R. Germany) for 45 min and Reynold solution (Lead nitrate and sodium citrate) around 1 h. Finally, the samples were observed with a Philips 208 S transmission EM at X resolution. The photographs were captured by Olympus digital camera (model E-20 p, 5M.P).

### Statistical analyses

The data resulting from the present study were analyzed by χ^2^-test. Associations were statistically significant when a *P*-value of less than 0.05 was observed.

## Results

### Blood smears

**Fig. 1:**
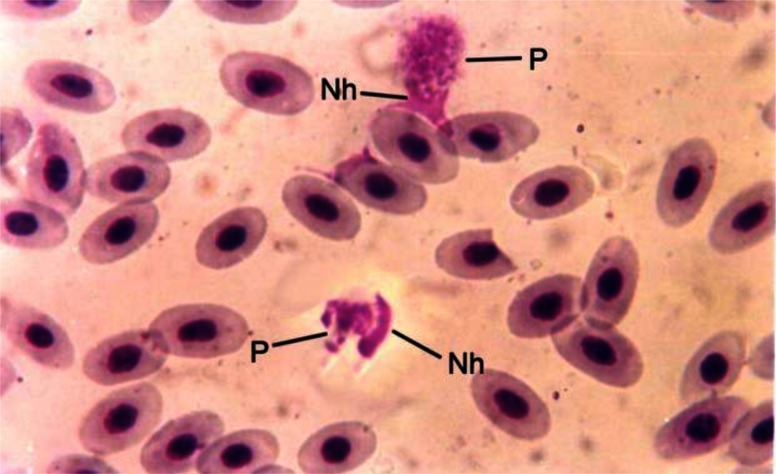
Duck blood smear; gametocytes of *leucocytozoon* spp. (P) located in red or white blood cells. The host cell nucleus (Nh) is pushed to one pole (Giemsa stain; Magnification 1000×)

Examination of 825 thin blood smears taken from bird's adults or chicks from the northern, central and southern of Lorestan province revealed evidence of *Leucocytozoon* species parasitaemia of leukocytes and red cells by light microscopy (Fig. 1).

Two hundred seventy-five birds were examined from Lorestan province, of which 44 (16.0%) were infected with *Leucocytozoon* species. Birds with relatively prevalence of *Leucocytozoon* included the chicken with 5.1% (n = 14) infected, the geese with 4.3% (n = 12) infected, ducks with 3.6% (n = 10), and the turkeys with 2.9% (n = 8) infected (Table 1). Thirty-five percent (10 of 29) of the birds examined in the Noor Abad area were infected. The highest population of infected birds (52.4%) was located in the northern of Lorestan province. The frequency of infection was higher in the north area as compared to the birds in the other studied areas and this difference was significant (*P*<0.05).

**Table 1:** Prevalence of *Leucocytozoon* in domestic birds at collected different sites

Site	Number collected birds (%)				Total	Number infected birds (%)				Total	*P* *η*
	G*a*	C*b*	T*c*	D*d*		G	C	T	D		
**North** ^***e***^											<0.05
Broujerd	8 (9.2)	4 (4.0)	4 (8.0)	2 (5.2)	18 (6.5)	0 (0.0)	1 (7.1)	1 (12.5)	3 (300.)	5 (11.4)	
Alishtar	7 (8.0)	11 (11.1)	9 (18.0)	7 (17.9)	34 (12.4)	4 (33.3)	1 (7.1)	2 (250.)	1 (100.)	8 (18.2)	
Noor Abad	10 (11.5)	5 (5.1)	4 (8.0)	6 (15.4)	29 (10.5)	2 (16.7)	4 (28.6)	3 (37.5)	1 (100.)	7 (15.9)	
**Center**											
Khorram Abad	26 (29.9)	51 (51.5)	14 (28.0)	11 (28.2)	102 (37.1)	3 (25.0)	2 (14.2)	1 (12.5)	1 (100.)	7 (15.9)	<0.05
**South**											
Koh Dasht	11 (12.6)	8 (8.1)	15 (30.0)	6 (15.4)	44 (16.0)	3 (25.0)	3 (21.4)	0 (0.0)	2 (200.)	8 (18.2)	>0.05
Pole Dokhtar	25 (28.7)	20 (20.2)	4 (8.0)	7 (17.9)	56 (20.4)	0 (0.0)	3 (21.4)	1 (12.5)	2 (200.)	8 (18.2)	
**Total**	87 (100)	99 (100)	50 (100)	39 (100)	275 (100)	12 (100)	14 (100)	8 (100)	10 (100)	44 (100)	

a Geese

b Chicken

c Turkey

d Duck

η *Chi-square* test (*x^2^*)

### Pathological findings

**Fig. 2:**
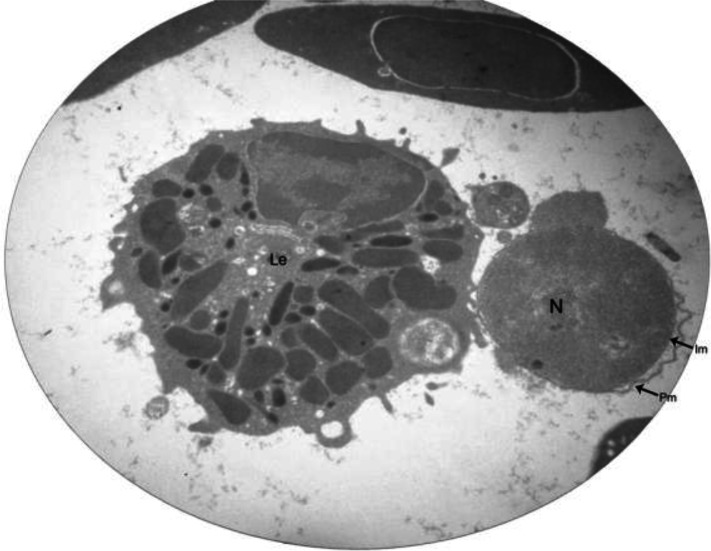
Transmission electron photomicrograph of an extracellular gametocyte with distinct membranes and nucleus (N). The outermost layer or pellicle membrane (Pm) is separated from inner membrane (Im). The leukocyte (Le) is on the left (Magnification 11000×)

The gametocytes were observed intra and extracellular in mononuclear cells and were of the ovoid type. The extracellular gametocytes were surrounded by distinct three-layered pellicle. The nucleus was observed in the gametocytes (Fig. 2).

**Fig. 3:**
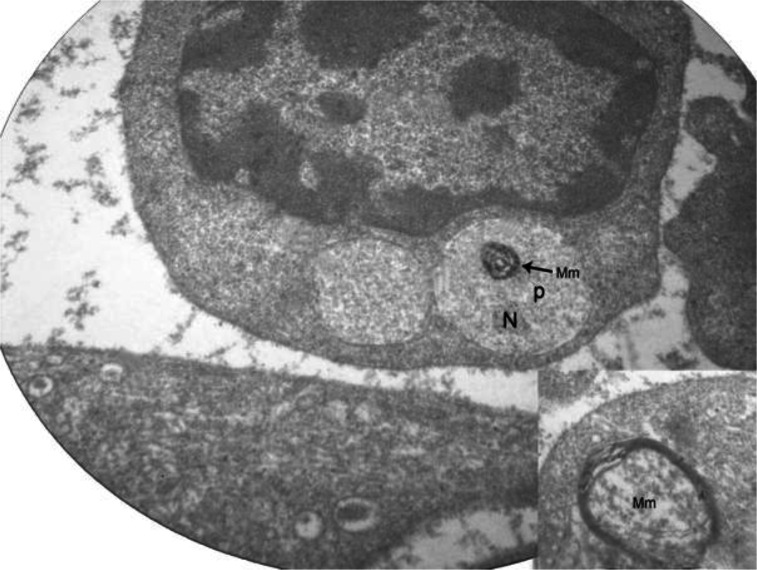
Transmission electron photomicrograph of immature stage of intracytoplasmic parasite (P) with nucleus (N) and myelin membrane structure (Mm) (Magnification 14000×). *Inset:* higher magnification of myelin membrane (18000×)

Intracellular parasites were also revealed inside the cytoplasm of the host cell and fully differentiated from the inner parts of the cell. Similar myelin-like structures were found within the cytoplasm of the parasites, a multi-concentric structure that seem associated with the inner parasite membrane (Fig. 3).

**Fig. 4:**
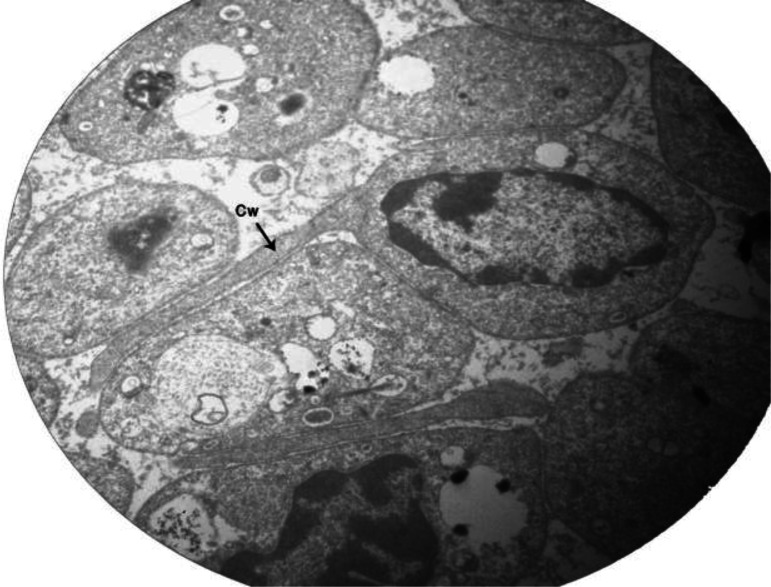
A cytoplasmic wing (Cw) is formed from host cell cytoplasm extension (Magnification 11000×)

The gametocytes had single nucleus with thinly dispersed chromatin surrounded by a membrane. The cytoplasmic wings were in the host cell including microfibriles that were near to the cytoplasmic membrane (Fig. 4).

## Discussion

There were numbers of reports on *Leucocytozoon* in many birds in Africa (3, 7, 8), Israel (9), New Zealand (4), Spain (10), and USA (11) and near areas like as Turkey (12, 13). However, this is the first study the prevalence and ultrastructural investigation of *Leucocytozoon* among domestic birds in Lorestan Province, southwest of Iran. The present data demonstrated that *Leucocytozoon* spp. infections are distributed in the study area birds’ population. The findings showed that 52.4% of infected birds were located in the north of the area.

Similar to *Plasmodium* and *Haemoproteus*, the exflagellation process of *Leucocytozoon* is a form of sexual maturation which takes place in the midgut of the insect vector host. With the release from host cell, the microgametocyte became underwent a process of maturation with the formation of microgametocyte and subsequent exflagellation (14). The factors including surface tension, temperature change, mechanical force and pH were listed as being responsible for the rupture of host cell membrane. A previous study revealed that exflagellation of mature microgametocytes took a place in a moist environment within 2.5 to 3 minutes (15). The myelin-like membrane proliferations were either associated with the inner parasite membrane or were isolated in the gametocyte cytoplasm, which appeared as double or triple membranes or even as whip-like whirling investigations. It seems that this increase in membrane surface could help to extending the absorptive or excretory capabilities of the parasite. These structures represented a proliferation of the host cell membrane lining the parasitophorous vacuole. An ultrastructural study showed that these membrane proliferations could be in many of the parasites examined, particularly in the more mature forms. These finding, combined with the fact that they were never found in normal uninfected lymphocytes, indicated that these myelin-like membrane proliferations were a true structure whose function is unknown (2).

The triple-layered pellicles of the parasite support the view that the outermost membrane is probably established by the host. In the early stages of infection, all three membranes were smooth and continuous with feeding probably occurring via diffusion. As the parasite is more mature, the outer membrane became less continuous and revealed to be dissolving with the space between the membranes becoming larger and continuous. Desser et al. (1987) referred to the microfibrils extending to the “cytoplasmic wings” of mature gametocytes as being microtubular struts which may serve a supportive function in maintaining the shape and rigidity of *Leucocytozoon* in infected host cells (16).

## Conclusion

This study is the first detailed description of the ultrastructural development of *Leucocytozoon* spp. in our area. Molecular surveys with other morphological and ecological findings could be useful for identifying the species. The results demonstrate parasitized domestic birds with *Leucocytozoon*. A long term study within the bird population is essential in order to disclose seasonal variation in parasite prevalence, and age of infection in the area.
